# Possibility of Using Surgical Pleth Index in Predicting Postoperative Pain in Patients after Vitrectomy Performed under General Anesthesia

**DOI:** 10.3390/diagnostics14040425

**Published:** 2024-02-14

**Authors:** Michał Jan Stasiowski, Anita Lyssek-Boroń, Magdalena Kawka-Osuch, Ewa Niewiadomska, Beniamin Oskar Grabarek

**Affiliations:** 1 Chair and Department of Emergency Medicine, Division of Medical Sciences in Zabrze, Medical University of Silesia, 40-555 Katowice, Poland; 2 Department of Anaesthesiology and Intensive Care, 5th Regional Hospital, Trauma Centre, 41-200 Sosnowiec, Poland; 3 Department of Ophthalmology with Paediatric Unit, 5th Regional Hospital, 41-200 Sosnowiec, Poland; anitaboron3@gmail.com (A.L.-B.); mkawka.buszman@gmail.com (M.K.-O.); 4 Department of Ophthalmology, Faculty of Medicine in Zabrze, Academy of Silesia, 40-555 Katowice, Poland; 5 Department of Epidemiology and Biostatistics, School of Public Health in Bytom, Medical University of Silesia, 40-555 Katowice, Poland; e.j.niewiadomska@gmail.com; 6 Faculty of Medicine, Collegium Medicum, WSB University, 41-300 Dabrowa Gornicza, Poland; bgrabarek7@gmail.com

**Keywords:** nociception/anti-nociception monitoring, adequacy of anesthesia, surgical pleth index, vitreoretinal surgeries, intolerable postoperative pain perception

## Abstract

Adequacy of anesthesia concept (AoA) in the guidance of general anesthesia (GA) is based on entropy, and it also reflects the actual depth of anesthesia and the surgical pleth index (SPI). Therefore, this study aimed to analyze the potential existence of relationships between SPI values at certain stages of the AoA-guided GA for vitreoretinal surgeries (VRS) and the incidence of intolerable postoperative pain perception (IPPP). A total of 175 patients were each assigned to one of five groups. In the first, the VRS procedure was performed under GA without premedication; in the second group, patients received metamizole before GA; in the third, patients received acetaminophen before GA; in the fourth group, patients received Alcaine before GA; and, in the peribulbar block group, the patients received a peribulbar block with a mix of the solutions of lignocaine and bupivacaine. Between the patients declaring mild and statistically significant differences in the IPPP in terms of SPI values before induction (52.3 ± 18.8 vs. 63.9 ± 18.1, *p* < 0.05) and after emergence from GA (51.1 ± 13 vs. 68.1 ± 8.8; *p* < 0.001), it was observed that the patients postoperatively correlated with heart rate variations despite the group allocation. The current study proves the feasibility that preoperative SPI values help with predicting IPPP immediately after VRS under AoA guidance and discrimination (between mild diagnoses and IPPP when based on postoperative SPI values) as they correlate with heart rate variations. Specifically, this applies when the countermeasures of IPPP and hemodynamic fluctuations are understood to be of importance in reducing unwelcome adverse events.

## 1. Introduction

Vitreoretinal surgeries are very common operations in ophthalmology. They are currently performed either under regional anesthesia (RA) with mild sedation or under general anesthesia (GA), based on the patient’s individual needs or anesthetic regimen in specific medical centers [1]. The guidance regarding GA in the elderly with comorbidities constitutes a challenge in view of the possible complications that could occur in the postoperative period as a result of inappropriate anesthesia administration and/or analgesia administration; as such, the digital monitoring of the abovementioned GA components is becoming increasingly popular [2,3].

Adequacy of anesthesia concept (AoA) in the guidance of GA is based on entropy electroencephalography (EEG), which reflects the actual depth of the anesthesia administration and the surgical pleth index (SPI, GE Healthcare, Helsinki, Finland; mathematically, SPI = 100 − (0.33* normalized heartbeat interval (HBInorm) + 0.67* pulse photoplethysmographic amplitude (PPGAnorm)). Such a calculation is provided by the manufacturer, and the SPI value is automatically calculated by the AoA software (NMT monitoring; Carescape B650, GE, Helsinki, Finland); as such, it does not need to be manually calculated. Rather, it is displayed online on a monitor interval (HBInorm and PPGAnorm). Therefore, it is similar to the calculation of the Bispectral index in that it constitutes business confidentiality, as the formula is not publicly revealed by the manufacturer [4]. Instead, it is derived from the normalized HBInorm and PPGAnorm from the pulse oximetry measurements, and it has also been proposed as a type of anti-nociception/anti-nociception balance during surgical procedures under GA [5]. The SPI is employed as a valuable tool to facilitate the precise titration of intraoperative opioid analgesics in vitreoretinal surgeries (VRS) [6]. The rationale behind utilizing the SPI in this context stems from the critical role of maintaining optimal intraoperative analgesia. Inadequate analgesia during the surgical procedure poses a considerable risk as it can potentially culminate in the development of intolerable postoperative pain perception (IPPP). The significance of preventing IPPP is underscored by its potential repercussions, including the likelihood of delayed recovery [5] and extending the length of hospital stay [7].

The consequences of inadequate intraoperative analgesia reach beyond simple discomfort, as suggested by this study [7]. It can also indicate a connection between insufficient pain management and increased risks of morbidity and mortality [7].

This association involves the initiation of cardiovascular and pulmonary complications, which are amplified when postoperative pain receives inadequate attention [8]. Consequently, the utilization of an SPI-guided titration of intraoperative opioid analgesics is presented as a pre-emptive strategy to alleviate the potential hazards linked with inadequate pain control. The objective was to reduce postoperative complications, accelerate the recovery process, and enhance the overall patient outcomes within the realm of vitreoretinal surgeries [9,10,11,12,13].

This study hypothesizes that monitoring the depth of anesthesia when using the AoA concept, specifically through the SPI and during VRS under GA, can be correlated with the incidence of IPPP. This research aims to explore whether SPI values at different stages of the AoA-guided GA for VRS are associated with the occurrence of IPPP. The hypothesis suggests that identifying and addressing SPI variations during specific phases of the procedure may enable proactive measures so as to prevent IPPP-related complications, such as delayed recovery, extended hospital stay, and increased risks of morbidity and mortality. This study also considered the potential impact of the SPI-guided titration of intraoperative opioid analgesics on IPPP as well as aimed to contribute insights into optimizing analgesic strategies to enhance perioperative outcomes and patient satisfaction in the context of VRS.

Therefore, the purpose of this study was to investigate the relationship between SPI values at specific stages of the AoA-guided GA during VRS and instances of IPPP.

## 2. Materials and Methods

This study was built upon the work conducted in our previous papers [14,15].

### 2.1. Ethical Considerations

In compliance with the guidelines in the Helsinki Declaration, ethical approval for this study (KNW/0022/KB1/101/15) was provided by the Ethical Committee of Medical University of Silesia on 29 September 2015 (Chairman Ph. Dr. Maria Trusz-Gluza). The project was registered in the Clinical Trial Registry (SilesianMUKOAiIT2, NCT02973581). Written informed consent was obtained from each patient. The patient information was securely archived in compliance with the relevant regulations at the Department of Anesthesiology and Intensive Therapy of the 5th Regional Hospital in Sosnowiec, Poland. Randomization was conducted through the unveiling of sealed envelopes following the receipt of written informed consent. To ensure confidentiality, patient data were removed before initiating the analysis, thereby preventing the identification of individual patients.

### 2.2. Study Design and Participants

Patients who were scheduled for elective vitreoretinal surgery in the Department of Ophthalmology at St. Barbara’s Memorial Hospital no. 5 in Sosnowiec, Poland, and met the inclusion criteria were asked to participate in the study. All vitreoretinal surgeries were performed by one vitreoretinal surgeon (A.L.-B.) using the same technique and the same vitreoretinal machine (Constellation, Alcon, Fort Worth, TX, USA). Pars plana vitrectomy is a frequently utilized method in vitreoretinal surgery, and it allows for controlled access to the posterior segment for the treatment of conditions such as retinal detachments, vitreous hemorrhage, endophthalmitis, and macular holes within a closed system. The procedure is named for the removal of vitreous (vitreous + ectomy = vitreous removal), with instruments introduced into the eye through the pars plana [14,15].

Two hundred patients with indications of vitrectomy were invited to participate in the study. The group size was determined by considering the annual total of vitrectomy procedures at the 5th Regional Hospital in Sosnowiec, Poland, which amounted to 333, with a confidence level of 95% and a margin of error of 5%. Ultimately, a total of 175 adult patients with an American Society of Anesthesiologists (ASA) score of I–III were enrolled after obtaining written informed consent.

Out of the 176 participants enrolled in this study, the conclusive analysis involved 175 individuals, comprising 97 (55.4%) women and 78 (44.6%) men. The subjects were distributed among five groups—the GA group (GA Group), metamizole group (M Group), acetaminophen/paracetamol group (P Group), peribulbar block group (PBB Group), and the topical anesthesia group (T Group)—with 40 (20%) patients in each group. A single participant was excluded from VRS due to an unexpected occurrence of heart rate disruptions that were accompanied by hypotension. Ultimately, each group consisted of 35 (20%) patients.

The exclusion criteria were as follows: pregnancy; drug or alcohol abuse; a history of neurological disease or a neurosurgical operation that would impair entropy EEG monitoring; a history of pulmonary disease, i.e., signs predicting difficult laryngeal mask placement; cofounding factors of a proven impairment of SPI monitoring; and the preoperative and intraoperative administration of drugs, such as beta-receptor blockers and vasoactive drugs (like atropine, ephedrine, the presence of a pacemaker, and cardiac arrhythmia) [16].

Patients were randomly assigned to one of five peer groups: (1) The GA Group (n = 35), which included patients receiving general anesthesia alone; (2) The T Group (n = 35), consisting of patients who received preventive topical analgesia (performed by M.K.) through a triple instillation of 2% proparacaine (Alcaine, propacaine hydrochloride ophthalmic solution, USP 0.5%, 15 mL, Sandoz a Novartis Company) 15 min before the induction of GA; (3) The PBB Group (n = 35), comprising patients who underwent peribulbar block (PBB) using a mixture of 3.5 mL of 2% lignocaine each (Lignocainum Hydrochloricum WZF 2% solution, 20 mg/mL, 2 mL, Polfa Warszawa S.A, Warsaw, Poland) and 0.5% bupivacaine (Bupivacainum Hydrochloricum WZF 0.5%, 5 mg/mL, 10 mL, Polfa Warszawa S.A, Warsaw, Poland) via Hamilton’s technique, which was administered 1 min before the induction of general anesthesia [17]; (4) The M Group (n = 35), comprising patients who received preemptive analgesia (PA) with a single dose of 1 g of metamizole (Pyralgin 0.5 g/mL, 5 mL solution; Polpharma SA, Starogard Gdanski, Poland) in 100 mL of a saline solution that was administered intravenously 30 min before arriving at the operating room; (5) The P Group (n = 35), comprising patients who received preemptive analgesia with a single dose of 1 g of acetaminophen (Paracetamol Kabi 10 mg/mL, solution 100 mL; Fresenius Kabi, Warsaw, Poland) in 100 mL of a saline solution that was intravenously administered 30 min before arriving at the operating room.

Acetaminophen was administered 30 min before the induction of GA, which was performed in this manner because its peak of action is approximately 45 min after intravenous administration. When you add the around 15 min needed for stages 1 and 2 at speculum installation, the action of the drug is at its peak and lasts for several hours later. PBB was performed before the induction of GA because we intended to achieve only sensory block. Furthermore, as we next conducted GA, global akinesia was not needed. Topical anesthesia was performed 15 min before the induction of GA in the majority of cases regarding venous placement performance.

Irrespective of the assigned group, all of the patients received oral premedication on the day of surgery in the form of 3.75–7.5 mg midazolam (Dormicum Midazolam 7.5 mg, Roche Polska sp. z o.o., Warsaw, Poland), which was administered 45 min prior to the induction of anesthesia, with the dosage being determined based on body weight and age [18].

### 2.3. Stages of AoA

#### 2.3.1. Stage 1

On admission to the operating theater, an entropy EEG (RE, SE) sensor was utilized on the patient’s forehead; a pulse oximeter (SPI) was used on the contralateral finger to facilitate venous access and access the NIBP cuff on the right arm; and a standard electrocardiography (ECG) was utilized on the patients’ back and was placed according to the manufacturer’s suggestions, where the first values were recorded. Directly before VRS, the patients were preoxygenated for 5 min with 100% oxygen and 10 mL/kg per body weight of a Ringer Solution was infused intravenously. Anesthesia was induced intravenously with 1 mcg/kg body weight of fentanyl, and etomidate (Etomidate Lipuro, Braun, Melsungen, Germany) was then administered at a single dose of 0.2–0.3 mg/kg of body weight to achieve a target state entropy (SE) around 40. After loss of consciousness, the patients in all groups were paralyzed with a standard intravenous dose of 0.6 mg/kg rocuronium (Esmeron, Fresenius, Warsaw, Poland); then, a laryngeal mask was placed after 45 s.

#### 2.3.2. Stage 2

SPI values were taken into account, starting from 5 min, in order to calculate the mean SPI value during Stage 2, and this was conducted after laryngeal mask placement and prior to the beginning of the sterilization of the orbita to allow for the calibration of the SPI sensor. After the laryngeal mask was installed, the SPI value was observed during preoperative preparations, which lasted at least 15 min, in order to calculate the SPI baseline. Then, the etCO_2_ levels were maintained at 35–37 mmHg, and the sevoflurane concentration was also maintained at the level of around 40–45 state entropy.

#### 2.3.3. Stage 3: Intraoperative

As the accuracy of nociception scores has been proven to be influenced by the GA regimen [15], all of the patients were anesthetized using a strict protocol where even changes in position on the operating table were accounted for [16]. The SPI score was monitored online and recorded with a sampling frequency of 1 min, which meant that the SPI was continuously observed in real-time during the specified medical procedures. The monitoring process involved assessing the SPI score at regular intervals, specifically every 1 min. The SPI score, derived from various physiological measurements, provides insights into the depth of anesthesia during surgery. The use of a 1-min sampling frequency indicated that the data points for the SPI score were collected at one-minute intervals throughout the duration of the procedures, thereby allowing for a detailed and dynamic assessment of the anesthesia depth. When the SPI value reached an ∆SPI of >15 points above the mean SPI value of Stage 2, a rescue dose of 1 mcg/kg body weight of fentanyl (FNT) was administered intravenously every 5 min until the SPI value decreased to value of the mean for SPI Stage 2. Throughout the AoA-guided GA for VRS, standard monitoring procedures were utilized, and close attention was paid to vital parameters such as non-invasive arterial pressure (BP), heart rate (HR), standard electrocardiography (ECG) II, arterial blood saturation (SaO_2_), the fraction of inspired oxygen in the gas mixture (FiO_2_), the fraction of inspired sevoflurane (FiAA), the fraction of expired sevoflurane (FeAA), the exhaled carbon dioxide concentration (etCO_2_), and the minimal alveolar concentration of sevoflurane (MAC). The depth of anesthesia was monitored with an entropy EEG (i.e., state and response entropy).

Similar to the methodology of inadequate hemodynamics [17], all of the studied groups were defined as follows: hypotension (mean arterial pressure; MAP < 65 mm Hg), hypertension (MAP > 110 mm Hg), bradycardia (HR < 45 beats min^−1^), or tachycardia (HR > 100 beats min^−1^). The treatment for inadequate hemodynamics included the infusion of a crystalloid solution (5 mL kg^−1^ Optylite), 10 mg of urapidil (if hypertension was still persistent after three fentanyl bolus doses (1 µg/kg) within 15 min), and atropine (if the HR did not raise above 45/min after incidence of the oculocardiac reflex (OCR)). The time duration of VRS was counted from the speculum installation to the speculum removal.

#### 2.3.4. Stage 4: The Emergence from GA

The stage of emergence from GA was defined as time from speculum removal to laryngeal mask removal. After speculum removal, the administration of sevoflurane was discontinued, the fraction of inspired oxygen in the gas mixture (FiO_2_) was increased to 95%, and the fresh gas flow was increased above the patients’ minute ventilation volume to wash out sevoflurane. The standardized determination of the emergence from GA was as follows: an SE of >86; spontaneous ventilation providing an SaO_2_ of >92%; an etCO_2_ of <40; and the ciliary reflex and general responsiveness recurring. After this, the laryngeal mask was removed. Then, the patients were transferred to the Post-Anesthesia Care Unit (PACU).

#### 2.3.5. Stage 5: Postoperative

Standard institutional postoperative care in the PACU, which was covered via further monitoring by the anesthesiologic team, blinded the patient group allocation. Along with the postoperative hemodynamic parameters, the presence of adverse effects such as nausea, postoperative nausea and vomiting (PONV), and allergic reactions were monitored for each patient at the same time. In the case of PONV, ondansetron (Ondansetron Accord, Accord Healthcare Limited, Devon, UK) was administered intravenously in a single intravenous dose of 4 mg. An Optilyte solution was infused at 5 mL/kg body weight in the case of an MAP of <65 mmHg similar to what was performed during Stage 3. The patients received oxygen at 3 L/min via the nasal cannula. The patients were asked to record the intensity of their pain using the Numeric Pain Rating Scale (NRS), which ranges from 0 (no pain) to 10 (maximum pain) every 10 min. In the case of pain perception with an NRS of >3, a standard dose of non-steroid anti-inflammatory drug was administered intravenously, which was delivered in accordance with the contemporary guidelines of acute pain treatment issued by the Polish Society of Anaesthesiology [19]. The SPI values were monitored online, and the mean SPI values were recorded with a sampling time of 1 min (as per the trends detailed in software provided by the producer). The NRS and SPI values were recorded for acute pain (an NRS of 7–10), average pain (an NRS of 4–6), and mild pain (an NRS of 0–3) perception intervals. The patients were observed and monitored in the PACU from 15 to 30 min until transfer to the Department of Ophthalmology, albeit only if the Aldrete score at discharge from the PACU was 10 [18]. The monitoring and data recording were then ceased. As the sympathovagal balance was proven to be strongly influenced by the awake patients’ arousal and emotions, the patients in the PACU were observed when they were in conditions that were free from any potential environmental stressors, which thus helped to create comparable conditions for the observance of the SPI values between subjects. In each case regarding patient arousal, such as coughing, sneezing, attempt of position change to the side, etc., the data of the SPI values were not included in the final calculations.

### 2.4. Statistical Analysis

Statistical calculations were performed using STATISTICA 13.3 (Stat Soft, Cracow, Poland) and R package 3.1.2, GNU General Public License. The measured data were characterized using the mean and standard deviation X ± SD, as well as the median with an interquartile range of M (IQR). We used numbers and percentages and a test of the equality of proportions for the nominal data. The normality of distribution was checked with the Shapiro–Wilk W test. The significance of differences between the means was tested using the Student’s t-test or ANOVA test for multiple groups. The compatibility in the groups for skewed distributions was examined using the U Mann–Whitney test or the Kruskal–Wallis test by ranks. Similarly, in the multivariate analysis, a two-way ANOVA or the permutational ANOVA (aovp ANOVA, lmPerm package, R) tests were applied, which were used to examine the combined effect of the used analgesia and pain intensity. Additionally, the post hoc tests were run to confirm the differences between the groups. Statistical significance was set at the level of *p* < 0.05.

## 3. Results

The study included 175 patients, of which there were 97 (55.4%) women and 78 (44.6%) men. The patients were divided into the following five groups, which contained 35 patients (20%) each: GA, M, PBB, P, and T. Two patients (one allocated to the T Group and one allocated to the P Group) were excluded from the final analysis due to their inability to declare their postoperative pain perception. The detailed characteristics of the patients’ anthropometric data are shown in Table 1. No significant differences in the individual groups in the case of characteristics, i.e., age, height, weight, and BMI, were registered.

Significantly higher FNT values were registered among the patients from M Group (Table 2). Only three patients were declared as possessing an acute postoperative pain perception, two patients in the T Group and one patient in P Group. Therefore, further analysis involved assessing the tolerable (an NRS of ≤3) and intolerable (an NRS of 4–10) pain perception. In the study groups, the percentage of patients with postoperative intolerable pain perception ranged from 14.3% in the PBB Group to 23.5% in the T Group. There were no statistically significant differences in the individual groups with respect to the level of pain intensity on the NRS scale. The use of different preventive analgesia techniques did not affect the incidence of the negative postoperative reaction regarding the OCR (*p >* 0.05). PONV was significantly the most common in the T Group (*p* < 0.05).

**Figure 1:**
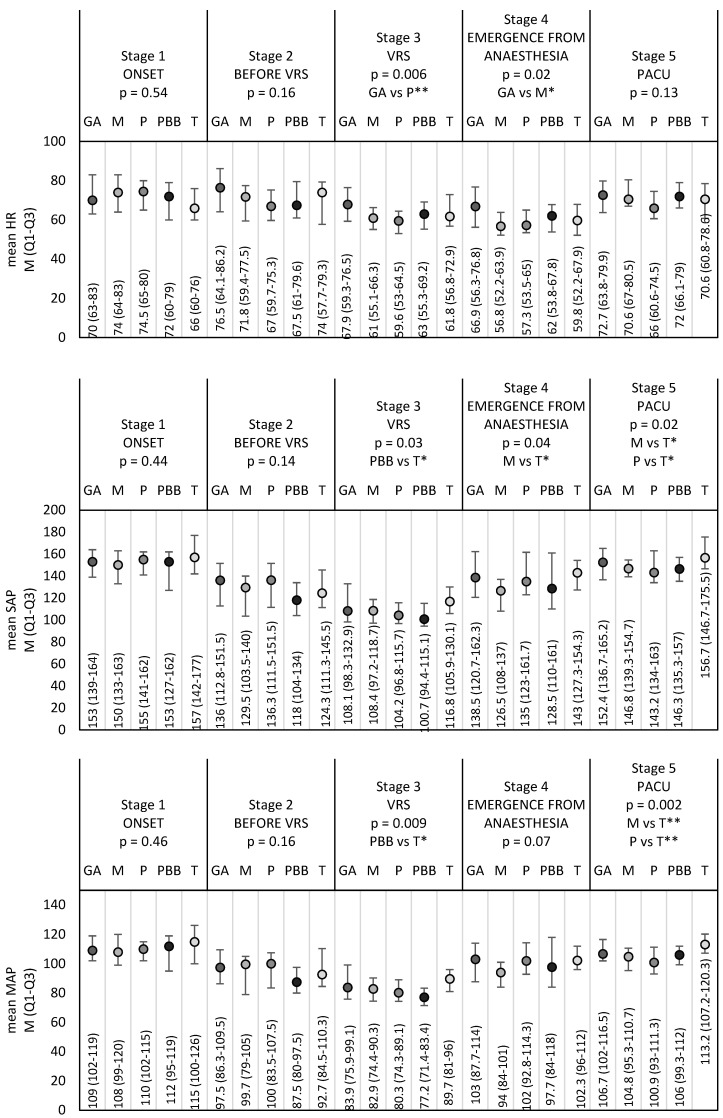

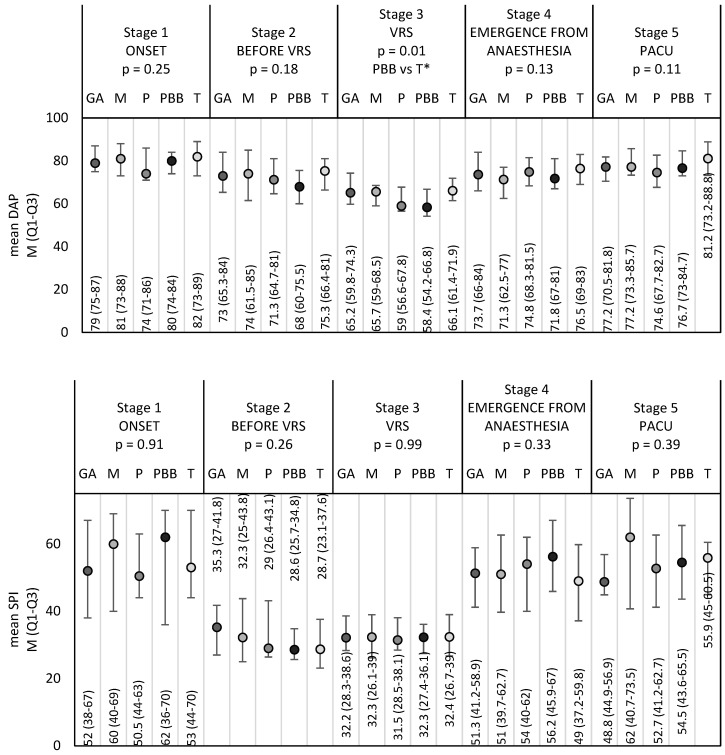

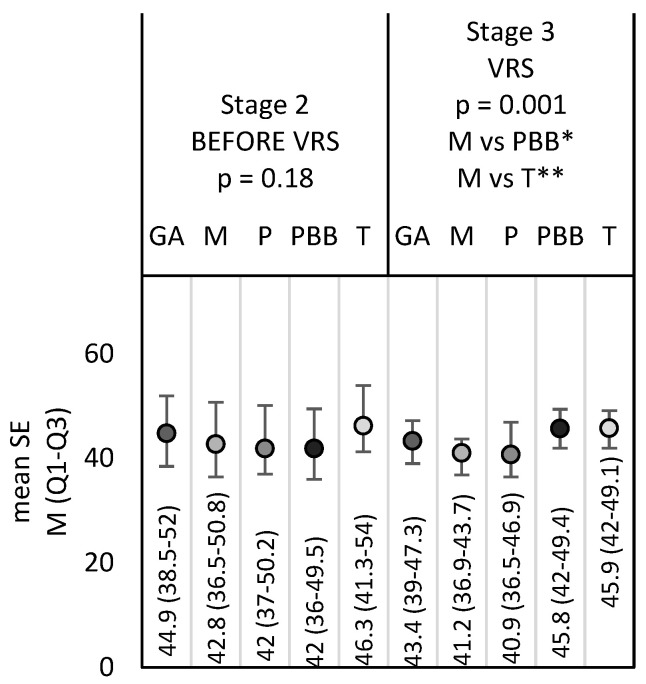
Comparison of the monitored patient parameter mean values (M (IQR)) at the same stage between the studied groups. The *p*-values were determined via one-way ANOVA or the Kruskal–Wallis test. The following symbols are defined thusly via post hoc tests: *—*p* < 0.05 and **—*p* < 0.01. Abbreviations: M, median; Q1, down quartile; Q3, upper quartile; HR, heart rate; VRS, vitreoretinal surgeries; SAP, systolic arterial pressure; PACU, Post-Anesthesia Care Unit; MAP, mean arterial pressure; DAP, diastolic arterial pressure; SPI, surgical pleth index; SE, state entropy; GA Group, the general anesthesia group; M Group, the metamizole group; P Group, the paracetamol group; PBB group, the peribulbar block group; and T Group, the topical anesthesia group.

The influence of pre-emptive intravenous analgesia on the mean hemodynamic parameter levels in the individual stages of the study is presented in Figure 1. There were no significant differences found in the baseline values (Stage 1) and values of the second stage (before VRS) in any of the groups. It was observed that, in the T group during VRS, there were significantly higher values of the following parameters: SAP (Systolic Arterial), MAP (Mean Arterial), DAP (Diastolic Arterial) and SE (Surgical Pleth Index). In the PBB group, however, there was a significantly higher level of SE values, while in the case of HR, significant differences were found in the GA and P Groups. Furthermore, significantly higher HR values were registered in the GA Group, while significantly higher SAP values were found in the T Group in Stage 4. Significant differences were observed in the case of the following parameters on the PACU Stage: SAP and MAP. No statistically significant differences were found in the mean SPI level in the studied groups at subsequent stages.

The higher level of pain (an NRS of 4–10) among all of the patients was associated with the significantly higher values of the mean SPI at Stage 1 (onset) (65 (23) vs. 53 (26), *p* = 0.003) and Stage 5 (PACU) (68.7 (12.3) vs. 49.6 (18.6), *p* < 0.0001), as well as due to the significantly higher values of the mean HR at Stage 4 (emergence from anesthesia) (63.6 (13.1) vs. 58.7 (13.4), *p* = 0.03) and Stage 5 (PACU) (75.2 (14) vs. 69.6 (15.2), *p* = 0.03) when compared with mild pain (an NRS of ≤3). Significantly higher values of the mean SPI were reported among the patients with an NRS of 4–10 at Stage 1 in the P Group as well as at the PACU stage in the M and P Groups (Table 3). On the other hand, significantly higher values of the mean SPI were reported among patients with an NRS of ≤3 at Stage 3 in the M and P Groups. Furthermore, significantly higher values of the mean SPI were reported among patients with an NRS of 4–10 on the second, third, and fourth stages in the T Group. The mean values of SAP varied due to the type of analgesia administered and the pain intensity encountered at the PACU stage.

**Figure 2:**
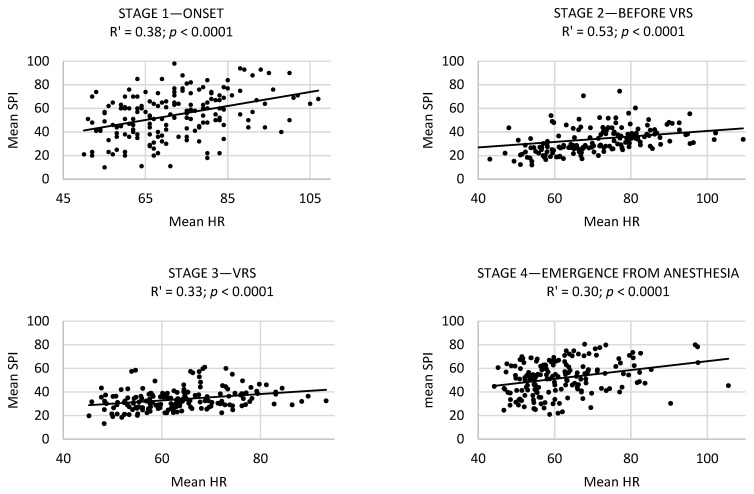

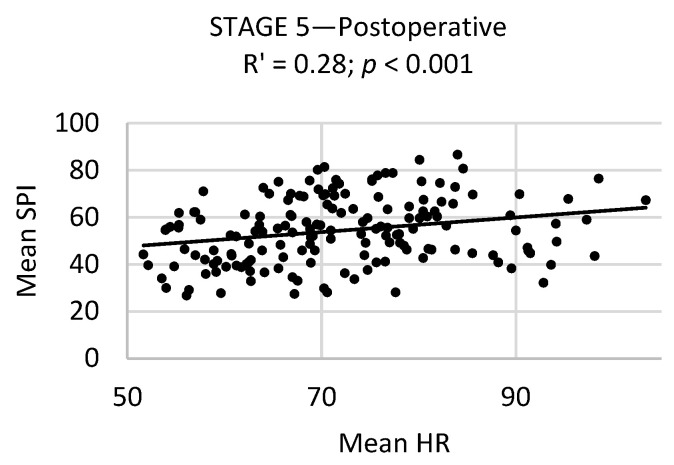
The strength of the correlation expressed by the Spearman correlation coefficient (R′) of the mean HR and SPI at Stages 1–5. Abbreviations: VRS, vitreoretinal surgery; PACU, post-anesthesia care unit; SPI, the Surgical Pleth Index and HR, heart rate.

The correlation analysis of the mean monitored patients’ parameters showed a significant positive correlation between the mean SPI and mean HR for all of the patients at every stage (Figure 2). This meant that, with the increase in the mean HR, the mean SPI increased significantly.

In the case of the group with higher pain levels (an NRS of 4–10), the correlation of the mean HR and SPI were at a similar level: onset: R = 0.38, p = 0.02; before VRS: R = 0.16, *p* = 0.38; VRS: R = 0.34, *p* = 0.052; emergence from anesthesia: R = 0.29, *p* = 0.09; postoperative: R = 0.3, *p* = 0.08. However, significant relationships were confirmed only at the beginning of the study (i.e., the onset stage). The significant, positive correlation of the mean SPI and mean DAP (R = 0.25, *p* < 0.05), or the mean HR (R = 0.38, *p* < 0.05), was observed during the first stage (onset) of the study. Moreover, the mean SPI values significantly correlated with the mean SAP, mean MAP, and mean DAP values at the second (before VRS) (where R was at the levels of 0.31, 0.36, 0.40, and *p* < 0.05, respectively) and fourth stages (emergence from anesthesia) (where R was at the levels of 0.35, 0.38, 0.43, and *p* < 0.05, respectively).

Significant differences in the averaged extreme hemodynamic parameters of HR and SPI were observed in the examined groups (Table 4). The significantly higher level of pain (an NRS of 4–10) among all of the patients was associated with higher values of the min SPI (62 (13) vs. 42 (19), *p* < 0.0001) and max HR (80 (14) vs. 76 (16), *p* = 0.02) at the PACU stage. There were no statistically significant differences in the averaged extreme values of the SPI between the studied groups at the next stages. In the case of the min HR, significantly higher values were reported among the patients with an NRS of 4–10 at the second, third, and fourth stages in the T Group. The averaged values of the min SPI and max SPI varied due to the pain intensity at the PACU Stage—higher values were reported in the group of patients with higher levels of pain (an NRS of 4–10).

## 4. Discussion

Promising future medical service providers, in the instruction of university medicine courses, should teach pain-relieving strategies [1,4] with simulation techniques in order to ensure that patients can enjoy freedom from intolerable pain. Delivering this would safeguard a basic human right, i.e., mitigating intolerable pain. As pain is, in its essence and definition, an ‘unpleasant sensory and emotional experience associated with actual or potential tissue damage or described in terms of such damage’ [5], the main problem seems to be a discrepancy between the assessment of the quality of pain perception and the assessment of the efficacy of analgesia modalities. Quantifying pain can be challenging for certain patients as some individuals may face difficulties in accurately assessing their pain perception. Even with the use of straightforward scales such as the Numeric Rating Scale (NRS) or Visual Analog Scale (VAS) [20], certain patients may find it burdensome to express their pain accurately. Additionally, there is a potential for misinterpretation, wherein patients might attribute a general feeling of unwellness after surgery, such as reporting a sore throat related to intubation or laryngeal mask use, as immediate postoperative pain (IPPP). Conversely, there is a tendency for certain individuals to under-report IPPP, whereby they consider it a natural consequence of the surgical intervention. This highlights the complexity of pain assessment and the need for a nuanced understanding of patients’ subjective experiences in the postoperative period. Some hope seems to reside in the emergence of possibilities regarding the digital monitoring of the nociception/anti-nociception balance, which is defined as a physiological encoding and processing of nociceptive stimuli [5]. The advancement of medical technologies that digitally represent the balance between nociception and anti-nociception, such as the anti-nociception index, the SPI, pupillometry, or nociception level, has the potential to bring objectivity to the subjective nature of pain assessment. This could open the door to a continuous loop of pain score evaluation via the utilization of these medical devices. Feedback from these assessments could be utilized to implement pre-analgesia techniques and rescue analgesia algorithms. This process would be guided by monitoring the normalization of values displayed on the screens of the abovementioned monitors.

As there is a growing number of elderly patients who require immobilization on the operating table because of poor cooperation with operators, despite mild sedation under RA, the employment of AoA for VRS comes into hand (as we have proven in our prior published reports). GA alone may be often particularly associated with unwelcome complications, like PONV in the first 24 h after VRS as a result of intraoperative opioid rescue analgesia [7] or due to life-threatening hemodynamic disturbances that potentially result in cardiovascular incidences. In addition, this could also apply to the OCR due to insufficient analgesia, as well as to the perception of pain postoperatively due to lack of pre-analgesia. (Note: this was obtained in the current study by using intravenous or regional techniques. See Methodology Section.) Therefore, the use of intraoperative narcotic analgesics should be rational, but monitoring the efficacy of analgesia still remains a challenge during GA. Several studies have reported fewer unwanted events, reduced opioid consumption, and shorter emergence from anesthesia when opioid titration is based on SPI guidance [8].

The employment of SE values in the observations with a target range of 40–45 created comparable conditions for every subject in the current study. However, what constituted an even stricter protocol, when compared to the current literature, was using the bispectral index within the range of a bispectral index (BIS) level between 40 and 60 in all of the patients [11]. The SPI was derived from the photoplethysmographic waveform amplitude and the heart beat-to-beat interval, and this proved to reflect the nociceptive–anti-nociceptive balance. There have also been attempts to monitor pain perception postoperatively via the observance of the variations in SPI values. Intraoperative SPI values between 20 and 50, according to the study of Chen X et al. [12], have been recognized as the proper intraoperative nociception/anti-nociception balance. As such, in the current study, the SPI values during Stage 2 were recorded at a range of 34.1 ± 22.9 in the patients reporting no IPPP vs. 34 ± 8.7 in the patients reporting IPPP. This was also performed alongside the SPI values during Stage 3, which were noted at 34.4 ± 9.4 in the patients reporting no IPPP in comparison to the SPI values that were noted at a range of 30.9 ± 6.5.4 in the patients reporting no IPPP in both stages, and this was recorded with no statistically significant difference, which is a token of the proper performance of the AoA-guided GA.

We identified high SPI values before the induction of GA as an independent risk factor for the incidence of IPPP. In the current study, despite the group allocation of the initial SPI value in the patients declaring a postoperative NRS of ≤ 3 in the PACU Stage, the SPI was 52.3 ± 18.8, whereas, in the patients declaring a postoperative NRS of 4–10 in the PACU Stage, the SPI was observed to be 63.9 ± 18.1, which was found to be statistically significant. We, therefore, observe that an increased sympathetic tone may predispose the patients to increased sensitivity to IPPP, which is burdensome with respect to quantification when only using the NRS. Our observations were rather surprising as no correlation between SPI values with stress hormones (ACTH, cortisol, epinephrine, norepinephrine) [21]—which play a significant role in the maintenance of the sympathetic tone [21]—has thus far been found during consciousness.

In the present study, we noted increased heart rate (HR) values at various stages of the general anesthesia (GA) in patients that reported the occurrence of immediate postoperative pain (IPPP) when undergoing vitreoretinal surgery (VRS), whether solely guided by a depth of anesthesia (AoA) alone or in conjunction with various pain management protocols. This was in comparison to the patients who reported a satisfactory postoperative pain experience. On the contrary, Hung K.C. et al. did not find correlations between elevated SPI values and hemodynamic parameters [22]. Gruenewald M. et al. [11] compared SPI-guided sevoflurane/sufentanil-based GA vs. GA-guided sevoflurane/sufentanil-based when using standard practice. They observed, in the SPI-guided group, an earlier and more pronounced increase in the SPI values in comparison with the HR values before the first application of sufentanil during surgery, and they thus concluded that the SPI could provide an earlier and even more pronounced and more sensitive method of detection for possible inadequate amounts of analgesia than would be ascertained via the observance of HR values alone. Wang M. et al. observed elevated SPI values under noxious stimulation via intubation incision, although it was not found to be predictive of the hemodynamic responses to intubation and incision [13], which is contrary to the current study’s findings.

It must be kept in mind that, with increasing age, the incidence of cardiovascular diseases increases, and monitoring analgesia via autonomic regulation can be altered due to neuropathy or the deterioration of the central nervous system [23]. Therefore, we speculate that, in patients undergoing VRS in whom co-morbidities like diabetes are frequent, these types of patients may be significant cofounders.

Studies in the current literature have reported the utility of observing SPI values to predict the incidence of IPPP [16]. Elevated SPI values at the end of surgery have proven useful for predicting the incidence of IPPP, which is defined as > 3 points when using the NRS [24]. A study was conducted on patients undergoing different surgical procedures, and it was underlined by the authors that this type of scenario was the most important limitation of their study findings [25]. In the current study, regardless of group allocation, there was no statistically significant difference in the mean, maximum, and minimum SPI values between the patients declaring IPPP and non-IPPP during emergence from the AoA-guided GA (Stage 4). Jung K et al. [26] observed the change in SPI values before and after skin incision in patients undergoing elective laparotomy under sevoflurane/fentanyl-based GA. They observed that an intraoperative SPI of >50 positively correlated with the incidence of IPPP. Moreover, they concluded that patients with observed increases in their SPI values at >23 after skin incision were positively correlated with excessive demands of postoperative opioid analgesics when compared to patients with lower increases in SPI values after skin incision. It was speculated that the aforementioned may play a key role in vulnerability to surgical noxious stimulation. Likewise, in the current study, the initial sympathetic tone expressed via SPI values prior to the induction of GA was found to be statistically significantly higher in patients with IPPP when compared to patients with mild postoperative pain perception, and this was the case despite the group allocation. Although Ledowski et al. have found that the observance of SPI value variations to be of no value for the assessment of pain in the PACU Stage [25], the current study findings have proven that investment in the AoA guidance of GA for patients undergoing VRS undoubtedly doubles the cost of anesthesiologic monitoring, as well as the cost of each GA administration due to the expense of disposable entropy sensors. As such, we would advise that monitoring SPI values is a good investment for predicting and assessing postoperative pain in patients undergoing VRS, though this is contrary to our observation concerning patients undergoing endoscopic procedures [6]. In the study performed by Lewandowski et al., the minimum and mean SPI values in the PACU Stage in patients declaring mild postoperative pain and incidences of IPPP (42.5 ± 13.2 vs.; 60 ± 9.2 and 51.1 ± 13 vs. 68.1 ± 8.8, respectively) were found to be statistically significantly higher when compared to patients declaring acceptable postoperative pain perception, and this was the case despite the group allocation in some of the studied groups [26].

Similar observations were made by Park M. et al. [27] in their post hoc analysis, as the cut-off for moderate-to-severe pain (an NRS of ≥5) was an SPI value of 60, whereas Thee C. et al. [28] reported that the observance of SPI values showed a moderate correlation with NRS scores in terms of discriminating between low and moderate pain (an NRS of ≤3), as well as between moderate and severe pain (an NRS of 7–10).

One should be aware of the fact that the utility of SPI observance in terms of the detection of incidence of IPPP in the PACU Stage can only be made under strictly defined circumstances [29]. As changes in intravascular fluid status, especially in fluid challenges, have already been proven to affect SPI readings [30], the analysis in the current study compared fluid challenges and found no statistically significant differences between the studied groups. Therefore, we concluded that the AoA guidance for GA with different PA regimens created comparable environments for individual subjects. Due to the autonomic tone from birth to senior age, patient age was found to change the values and thus served as another confounding factor [23]. Furthermore, no statistically significant differences were found in patients who were assessed for eligibility, thus proving the reliability of the current study’s outcomes.

Of course, our study has several limitations. First, the time duration of the emergence from GA (Stage 4) was, in some cases, only around several minutes. Therefore, only one measurement of the SAP, MAP, and DAP could be performed; as such, this might have affected the final results. Second, the postoperative pain perception in the PACU Stage might have been influenced by the intraoperative use of volatile anesthetics, IROA, and preoperative sedative agents, which may, therefore, have been mildly underreported. This could also apply, likewise, to the fact that pain intensity decreases with age. Third, as stated above, pain is a subjective phenomenon and some patients, even after proper training, fail to use the NRS properly. Fourth, we adopted a protocol where the indication for the administration of IROA was an ∆SPI of >15; however, this was in accordance with previous studies concerning the utility of the AoA guidance for GA in patients undergoing different surgical procedures [6]. We intended to avoid miscalculations in the case of low values of SPI and the possible overdosing of IROA. However, the employment of a stricter protocol—even though the standards in the current literature report that an ∆SPI of >10 or an SPI of >50 constitutes an indication for the administration of IROA—could have possibly resulted in hazardous opioid-induced bradycardia and hypotension, thereby potentially harming the patient [31]. Fifth, we did not observe correlations between the NRS and SPI values, nor between hemodynamic parameters, due to the reported influence of patient arousal with respect to the changes in |SPI values, as ascertained in the Department of Ophthalmology. Finally, an analysis of the risk factors of the incidence of IPPP in patients undergoing VRS under the AoA guidance was not reported in this paper, but it will be presented as a separate report.

## 5. Conclusions

In this study, we demonstrated that the observance of SPI values both for preoperative predictions and postoperative detections of IPPP in the PACU Stage in patients undergoing VRS under the AoA-guided GA being defined as an NRS of >3 was feasible and positively correlated with values of heart rate, thus indirectly confirming its reliability. Therefore, the employment of the AoA guidance of GA for improvements in the quality of postoperative care in patients undergoing specific surgical procedures requires further investigation for the sake of a more promising future for IPPP prevention policies, which will be possible due to introduction of instrumental techniques with respect to its monitoring and the objectivization of its efficacy in analgesic modalities.

## Figures and Tables

**Table 1:** The anthropometric data of the patients in the studied groups.

Anthropometric Data		Total n = 175	GA Group n = 35	M Group n = 35	P Group n = 35	PBB Group n = 35	T Group n = 35	*p*-Value
Age	(Years)	64.7 ± 11.7 66 (12)	65.1 ± 10.8 67 (9)	61.9 ± 11.9 63 (14)	66.6 ± 9.5 67.5 (8)	66.8 ± 12.1 69 (13)	62.9 ± 13.4 65.5 (14)	0.25
Gender	Female	97 (56.1)	18 (51.4)	15 (42.9)	24 (70.6)	21 (60)	19 (55.9)	0.21
	Male	76 (43.9)	17 (48.6)	20 (57.1)	10 (29.4)	14 (40)	15 (44.1)	
Height	(cm)	165.7 ± 8.7 165 (12)	166.9 ± 8.6 168 (14)	168 ± 7.4 170 (14)	162.9 ± 8.3 160 (11)	165.9 ± 8.3 164 (12)	164.6 ± 10.4 164 (18)	0.13
Weight	(kg)	77.7 ± 15.9 76 (17)	83.4 ± 19.8 82 (20)	74.7 ± 14.9 74 (19)	74.1 ± 13.5 72.5 (22)	78.8 ± 16 75 (11)	77.6 ± 13.6 80 (16)	0.18
BMI	(kg/m^2^)	28.3 ± 5.4 27.6 (6.2)	29.9 ± 6.6 28.4 (5.3)	26.4 ± 4.6 25.3 (5.4)	28 ± 5.4 27.7 (7.6)	28.6 ± 5.1 27.1 (4.4)	28.7 ± 4.8 28.4 (6.2)	0.15

Results presented as the mean ± SD and median (IQR). The *p*-values were determined via a one-way ANOVA test or Kruskal–Wallis test by ranks. Abbreviations: BMI, body mass index; SD, standard deviation; IQR, interquartile range; M Group, the metamizole group; P Group, the paracetamol group; PBB Group, the peribulbar block group; and T Group, the topical anesthesia group.

**Table 2:** Rate of the postoperative pain perception in the patients in terms of group allocation.

Scale		Total n = 175	GA Group n = 35	M Group n = 35	P Group n = 35	PBB Group n = 35	T Group n = 35	*p*-Value
Postoperative pain perception	Acute NRS (7–10)	3 (1.7)	0 (0)	0 (0)	1 (2.9)	0 (0)	2 (5.8)	0.23
	Moderate NRS (4–6)	30 (17.3)	6 (17.1)	8 (22.9)	5 (14.7)	5 (14.3)	6 (17.7)	0.88
	Intolerable NRS (4–10)	33 (19.1)	6 (17.1)	8 (22.9)	6 (17.7)	5 (14.3)	8 (23.5)	0.84
	Mild NRS (≤3)	140 (80.9)	29 (82.9)	27 (77.1)	28 (82.4)	30 (85.7)	26 (76.5)	0.84
Unable to assess postoperative pain perception in the		2	0	0	1	0	1	1.00
oculocardiac reflex (OCR)		20 (11.6)	4 (11.4)	6 (17.1)	4 (11.8)	4 (11.4)	2 (5.9)	0.69
Postoperative Nausea and Vomiting (PONV)		14 (8.1)	4 (11.4)	1 (2.9)	1 (2.9)	1 (2.9)	7 (20.6)	0.02 ^B^
NRS max		1.5 ± 2.1 0 (3)	1.5 ± 2 0 (3)	1.5 ± 2.2 0 (3)	1.6 ± 2.00 1 0 (3)	1.1 ± 1.9 0 (2)	1.8 ± 2.5 0 (3)	0.84
FNT requirement (mg)		128.2 ± 106.9 100 (150)	144.3 ± 102.7 150 (150)	165.7 ± 116.8 200 (200)	94.1 ± 82.4 100 (50)	95.1 ± 101.3 50 (150)	141.2 ± 113.8 125 (200)	0.02 ^A^
Intraoperative fluid challenge		991.8 ± 304.5 1000 (350)	955.7 ± 376.1 1000 (450)	1014.3 ± 334.4 1000 (500)	926.7 ± 196.4 1000 (250)	1075.7 ± 281.6 1000 (250)	976.8 ± 286.5 1000 (350)	0.16

Results presented as the mean ± SD and median (IQR) for the quantitative variables and numbers (percentages) for nominal variables. The *p*-values for the quantitative variables were determined via a one-way ANOVA test or the Kruskal–Wallis test by ranks. The ^A^-values were significantly higher (*p* < 0.05) in the M Group compared with values in the PBB Group; the *p*-values were ascertained via a test of equal proportions; and the ^B^-values were significantly higher (*p* < 0.05) in the T Group compared with the values in the M, P, and PBB Groups. Abbreviations: NRS, Numeric Rating Scale; SD, standard deviation; IQR, interquartile range; OCR, oculocardiac reflex; PONV, postoperative nausea and vomiting; FNT, fentanyl; M Group, the metamizole group; P Group, the paracetamol group; PBB group, the peribulbar block Group; and T Group, the topical anesthesia group.

**Table 3:** Comparison of the monitored patient parameter mean values (M (IQR)) with respect to the following metrics: SAP (mmHg), MAP (mmHg), DAP (mmHg), HR (beats/min), SPI, and SE. These metrics were assessed at the same stage in the studied groups with respect to postoperative pain (an NRS of ≤3 vs. an NRS of 4–10).

Parameter	GA Group		M Group		P Group		PBB Group		T Group		*p*-Value
	≤3	4–10	≤3	4–10	≤3	4–10	≤3	4–10	≤3	4–10	
STAGE 1—ONSET											
SAP	154 (19)	139 (6)	150 (32)	152.5 (21)	156.5 (26)	148.5 (30)	154.5 (35)	149 (30)	157 (48)	161.5 (42)	0.41
MAP	112 (16)	102 (7)	108 (23)	108.5 (12.5)	110 (17)	109.5 (9)	114 (24)	104 (12)	115 (22)	119 (24)	0.56
DAP	80 (12)	76.5 (4)	81 (16)	79.5 (9.5)	74 (15)	80 (16)	80 (13)	75 (3)	81 (16)	85.5 (11)	0.91
HR	72 (19)	67 (9)	70 (17)	82 (18)	75 (16.5)	73 (14)	70.5 (19)	73 (15)	66 (16)	65.5 (18.5)	0.99
SPI	52 (31)	61 (44)	60 (31)	60 (16.5)	50 (16)	75.5 (37) ^A^	58 (32)	70 (10)	52 (28)	67.5 (27)	0.91
STAGE 2—BEFORE VRS											
SAP	133.5 (37.5)	137.9 (41.5)	128 (44.5)	133.8 (21)	129.8 (44.3)	148.3 (11)	118.8 (31)	111 (19)	119.5 (40.5)	127 (15.5)	0.16
MAP	96.5 (22.3)	103.4 (30)	99.7 (30)	101 (18.5)	95.3 (26.9)	103.5 (7)	87.5 (21.5)	85 (11)	92.7 (26.3)	92.8 (19.3)	0.99
DAP	72.5 (17)	77.5 (22.5)	77 (28)	69 (17.3)	68.3 (14.3)	80.5 (7)	68.4 (17)	66 (7.3)	75.5 (14)	72.5 (17.1)	0.54
HR	77.3 (22.5)	69.7 (11.5)	71.8 (18.2)	70.5 (19.6)	66.8 (15.7)	70.9 (14.3)	68.6 (18.5)	65.3 (10.5)	67.9 (22.2)	80.8 (16) ^A^	0.17
SPI	36 (12.5)	32 (13.8)	34.8 (19)	30.8 (8.7)	28.9 (15.4)	39.8 (15.7)	28.4 (8.9)	33 (9.3)	27.5 (12.8)	34.7 (13.4)	0.75
SE	44.9 (12.1)	43.6 (15.5)	42.8 (14.3)	42.2 (17)	43.5 (12.2)	37.5 (5.2)	42.5 (13)	42 (14.7)	46.3 (12.7)	49.9 (13.2)	0.81
STAGE 3—VRS											
SAP	105.2 (37.5)	114.3 (9.4)	108.4 (22.2)	108.3 (21.1)	103.7 (22.1)	109 (12.9)	101 (15.6)	99.6 (28.6)	116.8 (26)	124.8 (24.7)	0.06
MAP	80.5 (24.5)	87.2 (4.6)	82.9 (16)	85.3 (12.1)	80.3 (15.8)	81.7 (10.5)	77.4 (11.8)	74 (16.7)	88.7 (14.2)	91.9 (21.8)	<0.05
DAP	64.4 (14.5)	67 (5.6)	65.3 (13.3)	66.2 (8.3)	59 (11.2)	61.6 (15)	57.1 (12.9)	62.2 (5.2)	63.6 (10.6)	70.8 (15.6)	<0.05
HR	70 (17.7)	63.9 (13.9)	61.9 (11.2)	59.4 (11.6)	58.8 (11.3)	62.3 (13.4)	63 (14.1)	63 (11.4)	59.6 (10)	74.1 (17) ^B^	<0.001
SPI	31.3 (9.4)	36.5 (10.3)	33.4 (10.2) ^C^	24.6 (12.7)	35.6 (13.6) ^C^	28.5 (4)	32 (8.1)	35.2 (7.3)	32.4 (12.3)	34.2 (10.3)	0.05
SE	43.4 (8.2)	43.3 (6.6)	39.4 (6.7)	42.9 (8.7)	40.9 (11.3)	41.6 (8.2)	46.1 (5.8)	43.9 (13.5)	46.3 (6.8)	45.3 (7.4)	<0.05
STAGE 4—EMERGENCE FROM ANESTHESIA											
SAP	138.5 (34.3)	129.6 (47)	123 (29.5)	134.5 (38.8)	139.8 (35.8)	124.5 (70)	131 (47)	110 (15.3)	139.8 (28.3)	147.3 (30.1)	0.09
MAP	103 (24)	99.9 (29.3)	93 (16)	99.3 (24.2)	102.3 (20.2)	95.8 (46)	98.5 (33)	84 (8.7)	100.5 (15.3)	109.8 (15.8)	0.09
DAP	72 (16)	78.6 (15)	71.3 (17.8)	72 (7.5)	74.3 (10.3)	84.3 (27)	73.5 (14.5)	68.7 (3)	74.8 (12.8)	82.2 (14.8)	0.06
HR	67 (19)	58.1 (14.9)	55.9 (11.1)	62.5 (14.6)	56.5 (12.5)	59.6 (15.9)	61.2 (14.3)	62.9 (6.2)	55.1 (10.6)	76 (15.3) ^A^	<0.0001
SPI	54 (15)	41.8 (26)	54.1 (23.2)	48 (18.4)	53.1 (20.4)	67.8 (25)	56.6 (21.1)	53.3 (20)	45.6 (26.1)	56.5 (15.2)	0.35
STAGE 5—POSTOPERATIVE											
SAP	157.6 (29.5) ^C^	135.4 (22.7)	146.3 (11.9)	150.8 (16.4)	140.5 (23.5)	165.1 (44.8)	146.7 (20)	141.3 (27.7)	154.4 (31.3)	160.8 (20.6)	<0.05
MAP	110.3 (17.5)	102.5 (8.7)	104.3 (15.3)	106.3 (16.2)	100.9 (15.2)	104.5 (31.4)	106.3 (11.9)	98.8 (14)	112.5 (13)	115 (15.9)	<0.05
DAP	77.2 (14)	77.6 (9.8)	77 (12.3)	78.3 (11.5)	72.5 (13.3)	77.7 (22)	77.3 (12.5)	75 (7.3)	81.2 (16)	83.3 (22)	0.17
HR	75 (17.3)	68.9 (9.7)	69.9 (8.9)	81.1 (11.1)	65.2 (13.3)	75.1 (18.5)	71.3 (15)	76.8 (12.5)	68.5 (17.9)	75.1 (9.8)	0.08
SPI	46.7 (11.3)	64.9 (16.7)	55.2 (31.5)	74.5 (8.2)^B^	49.4 (18.5)	70 (17) ^B^	53.3 (18.8)	69.2 (5.6)	53.2 (14.2)	62.1 (10.2)	<0.0001

Results are presented as the median (IQR. The *p*-values were determined via a two-way ANOVA test (with Group*NRS). The ^A^ significantly higher values (*p* < 0.05) for the group of patients with an NRS of 4–10 were determined via a post hoc test; the ^B^ significantly higher values (*p* < 0.01) for the group of patients with an NRS of 4–10 by the post hoc test; ^C^—significantly higher values (*p* < 0.05) for group of patients with an NRS of ≤3 were determined via a post hoc test. Abbreviations: NRS, Numeric Rating Scale; IQR, interquartile range; VRS, vitreoretinal surgeries; HR, heart rate; SAP, systolic arterial pressure; MAP, mean arterial pressure; DAP, diastolic arterial pressure; SPI, surgical pleth index; SE, state entropy; GA Group, the general anesthesia group; M Group, the metamizole group; P Group, the paracetamol group; PBB Group, the peribulbar block group; and T Group, the topical anesthesia group.

**Table 4:** Comparison of the hemodynamic fluctuation values (M (IQR)) of the following monitored patient parameters—HR (beats/min) and SPI—which was at the same stage between the studied groups due to postoperative pain (an NRS of ≤3 vs. an NRS of 4–10).

Parameter	GA Group		M Group		P Group		PBB Group		T Group		*p*-Value ^1^	*p*-Value ^2^
	≤3	4–10	≤3	4–10	≤3	4–10	≤3	4–10	≤3	4–10		
STAGE 2—Before VRS												
Max HR	85 (27)	76 (13)	76 (18)	81.5 (24)	73.5 (17)	72.5 (16)	75 (14)	67 (10)	75 (19)	84.5 (14)	0.10	0.09
Max SPI	41 (19)	41.5 (12)	42 (22)	39.5 (13.5)	35.5 (13.5)	45 (23)	36 (9)	41 (15)	37 (16)	38 (15.5)	0.15	0.52
Min HR	74 (19)	66.5 (11.5)	69 (16)	65 (19)	64 (14.5)	70 (16)	64 (18)	63 (11)	65 (21)	78 (16.5) ^A^	0.08	0.16
Min SPI	27 (11)	27.5 (14)	31 (16)	23.5 (8.5)	24 (10.5)	30 (16)	25 (12)	26 (1)	23 (12)	33 (11.5) ^A^	0.37	0.06
STAGE 3—VRS												
Max HR	81 (23)	82.5 (21)	72 (15)	79.5 (21)	67 (19.5)	74 (16)	72.5 (18)	75 (12)	71 (19)	96 (23.5) ^C^	<0.001 GA vs. M ** GA vs. P *** GA vs. PBB * GA vs. T*	<0.001
Max SPI	54.5 (21)	50.5 (7)	57 (19)	48 (18.5)	54.5 (15.5)	49.5 (16)	51.5 (18)	57 (1)	52 (21)	49 (18.5)	0.28	0.26
Min HR	63 (15)	58 (8)	56 (11)	49.5 (12)	52 (7.5)	55.5 (8)	57 (15)	51 (7)	51 (11)	65 (18.5) ^A^	<0.01 GA vs. P **	<0.05
Min SPI	23 (10)	18.5 (11)	21 (9) ^D^	14 (7.5)	22 (11) ^D^	17 (3)	19 (9)	20 (6)	20 (8)	24.5 (12)	0.42	0.05
STAGE 4—EMERGENCE FROM ANESTHESIA												
Max HR	77 (21)	63.5 (18)	61 (9)	67.5 (17.5)	61 (12)	71 (20)	69 (17)	67 (7)	60.5 (12)	82 (27.5) ^C^	<0.05 GA vs. M*	<0.0001
Max SPI	68 (17)	57 (24)	67 (18)	71 (17.5)	62 (12)	81.5 (25)	69 (16)	66 (13)	61 (28)	64.5 (13.5)	0.35	0.15
Min HR	61 (17)	56.5 (11)	54 (11)	59.5 (11.5)	53 (13.5)	56.5 (14)	57 (16)	59 (4)	52 (12)	66.5 (12.5) ^A^	0.10	<0.01
Min SPI	33 (21)	31.5 (24)	34 (32)	37 (20)	40 (22.5)	61 (35)	46 (28)	44 (14)	37 (30)	42 (17.5)	0.28	0.47
STAGE 5—PACU												
Max HR	79.5 (17)	75.5 (11)	74 (13)	86 (11)	69 (14)	85 (17)	78.5 (10)	79 (12)	71.5 (19.5)	80.5 (13.5)	0.08	<0.05
Max SPI	58 (12)	74.5 (11) ^B^	64 (30)	81.5 (12) ^B^	55 (16.5)	79.5 (19) ^B^	58.5 (15.5)	73 (12) ^A^	60 (16)	74.5 (10.5)	0.33	<0.0001
Min HR	69.5 (22)	64.5 (15)	67 (10)	75 (16)	62.5 (12)	68.5 (19)	65 (17)	73 (9)	63 (17.5)	71 (9)	0.32	0.32
Min SPI	38 (17)	57.5 (20) ^B^	43 (32)	63.5 (8) ^B^	43 (18)	64 (14) ^B^	45.5 (17.5)	64 (7) ^B^	43.5 (15.5)	52.5 (13.5) ^B^	0.14	<0.0001

Results are presented as the median (IQR). The 1-*p*-values were determined via a one-way ANOVA test or the Kruskal–Wallis test (by group). The 2-*p*-values were determined via a two-way ANOVA test or a non-parametric ANOVA test (by group * NRS). The ^A^ significantly higher values (*p* < 0.05) for the group of patients with an NRS of 4–10 were determined via a post hoc test; the ^B^ significantly higher values (*p* < 0.01) for the group of patients with an NRS of 4–10 were determined via a post hoc test; the ^C^ significantly higher values (*p* < 0.001) for the group of patients with an NRS of 4–10 were determined via a post hoc test; and the ^D^ significantly higher values (*p* < 0.05) for the group of patients with an NRS of ≤3 were determined via a post hoc test. Abbreviations: NRS, Numeric Rating Scale; IQR, interquartile range; VRS, vitreoretinal surgeries; PACU, Post-Anesthesia Care Unit; Min, minimum; Max, maximum; GA, the general anesthesia group; M Group, the metamizole group; P Group, the paracetamol group; PBB Group, the peribulbar block group; T Group, the topical anesthesia group; HR, heart rate; and SPI, the surgical pleth index. The following symbols are defined thusly via post hoc tests: *—p < 0.05, **—p < 0.01, and ***—p < 0.001.

## Data Availability

The data used to support the findings of this study are included within the article.
